# A nomogram model combining computed tomography-based radiomics and Krebs von den Lungen-6 for identifying low-risk rheumatoid arthritis-associated interstitial lung disease

**DOI:** 10.3389/fimmu.2024.1417156

**Published:** 2024-08-01

**Authors:** Nie Han, Zhinan Guo, Diru Zhu, Yu Zhang, Yayi Qin, Guanheng Li, Xiaoli Gu, Lin Jin

**Affiliations:** ^1^ Department of Ultrasound, Guanghua Hospital Affiliated to Shanghai University of Traditional Chinese Medicine, Shanghai, China; ^2^ Department of Radiology, Guanghua Hospital Affiliated to Shanghai University of Traditional Chinese Medicine, Shanghai, China; ^3^ Department of Pulmonary Function, Guanghua Hospital Affiliated to Shanghai University of Traditional Chinese Medicine, Shanghai, China

**Keywords:** computed tomography, radiomics, KL-6, rheumatoid arthritis, interstitial lung disease

## Abstract

**Objectives:**

Quantitatively assess the severity and predict the mortality of interstitial lung disease (ILD) associated with Rheumatoid arthritis (RA) was a challenge for clinicians. This study aimed to construct a radiomics nomogram based on chest computed tomography (CT) imaging by using the ILD-GAP (gender, age, and pulmonary physiology) index system for clinical management.

**Methods:**

Chest CT images of patients with RA-ILD were retrospectively analyzed and staged using the ILD-GAP index system. The balanced dataset was then divided into training and testing cohorts at a 7:3 ratio. A clinical factor model was created using demographic and serum analysis data, and a radiomics signature was developed from radiomics features extracted from the CT images. Combined with the radiomics signature and independent clinical factors, a nomogram model was established based on the Rad-score and clinical factors. The model capabilities were measured by operating characteristic curves, calibration curves and decision curves analyses.

**Results:**

A total of 177 patients were divided into two groups (Group I, n = 107; Group II, n = 63). Krebs von den Lungen-6, and nineteen radiomics features were used to build the nomogram, which showed favorable calibration and discrimination in the training cohort [AUC, 0.948 (95% CI: 0.910–0.986)] and the testing validation cohort [AUC, 0.923 (95% CI: 0.853–0.993)]. Decision curve analysis demonstrated that the nomogram performed well in terms of clinical usefulness.

**Conclusion:**

The CT-based radiomics nomogram model achieved favorable efficacy in predicting low-risk RA-ILD patients.

## 1 Introduction

Rheumatoid arthritis (RA) is one of the most immune-mediated diseases that affects 0.5–1% of the global population. It is primarily characterized by joint swelling and tenderness, leading to the destruction of synovial joints (1). Beyond the joints, RA is associated with systemic inflammation that can result in multiple coexisting conditions and extra-articular manifestations (2). Pulmonary involvement is recognized as the most prevalent extra-articular complication in RA, encompassing a broad range of disorders such as airway diseases, pleural effusions, and rheumatoid nodules (3–5). Among these pulmonary complications, interstitial lung disease (ILD) has the highest prevalence (6). Importantly, RA-ILD is a significant cause of mortality among RA patients and contributes to considerable morbidity (7). Consequently, accurately assessing mortality risk associated with RA-ILD is of great clinical significance.

The ILD-GAP (gender, age, and pulmonary physiology) index, initially proposed by Ley et al. in 2012 (8), is a simple scoring system designed to predict mortality risk in patients with idiopathic pulmonary fibrosis. Utilizing variables such as gender, age, predicted forced vital capacity (FVC), and diffusion capacity for carbon monoxide (DL_CO_), which has been refined and validated for various types of ILD (9). Its accuracy in predicting outcomes for RA-ILD has been confirmed by multiple studies (10–12). However, pulmonary function tests (PFTs) necessitate active participation from patients, such as performing deep breaths or forceful exhalations (13). This can be particularly challenging for special populations, including those with cognitive impairments or concurrent pulmonary conditions, potentially compromising the precision of the test results. To our knowledge, there is an absence of universal, quantitative, non-invasive techniques for the staging of RA-ILD.

The current primary method for diagnosing RA-ILD remains Computed Tomography (CT) scan, owing to its noninvasive and sensitive nature in detecting lung involvement (14, 15). However, there are many features to determine the presence of ILD and inter-reader variability, especially in unexperienced readers, is an issue (16). Visual analysis of ILDs on CT images faces difficulties in providing prognosis information, as various stages of RA-ILD exhibit overlapping imaging features, making the diagnosis and assessment of severity challenging with conventional imaging modalities (17, 18). Radiomics technology, capable of extracting numerous high-dimensional features from CT images, emerges as a solution to address the limitations of visual assessment. Although radiomics has predominantly been explored in the context of various tumors (19, 20), its potential has been demonstrated in identifying the GAP staging of connective tissue disease-associated interstitial lung disease (CTD-ILD) (21, 22). However, ILD associated with different CTDs can be characterized by distinct clinical manifestations, imaging, and pathological features, indicating their unique developmental and regression patterns. In the context of RA-ILD, evidence from a small cohort study suggested that radiomics may hold the potential for predicting mortality (23). However, limited studies are focusing on the application of radiomics in the staging of RA-ILD. Therefore, it is still necessary to explore the discriminative value of radiomics in various stages of RA-ILD.

In this retrospective study, we aimed to establish a novel CT-based radiomics nomogram to differentiate between the different stages of RA-ILD.

## 2 Materials and methods

### 2.1 Patients

**Figure 1:**
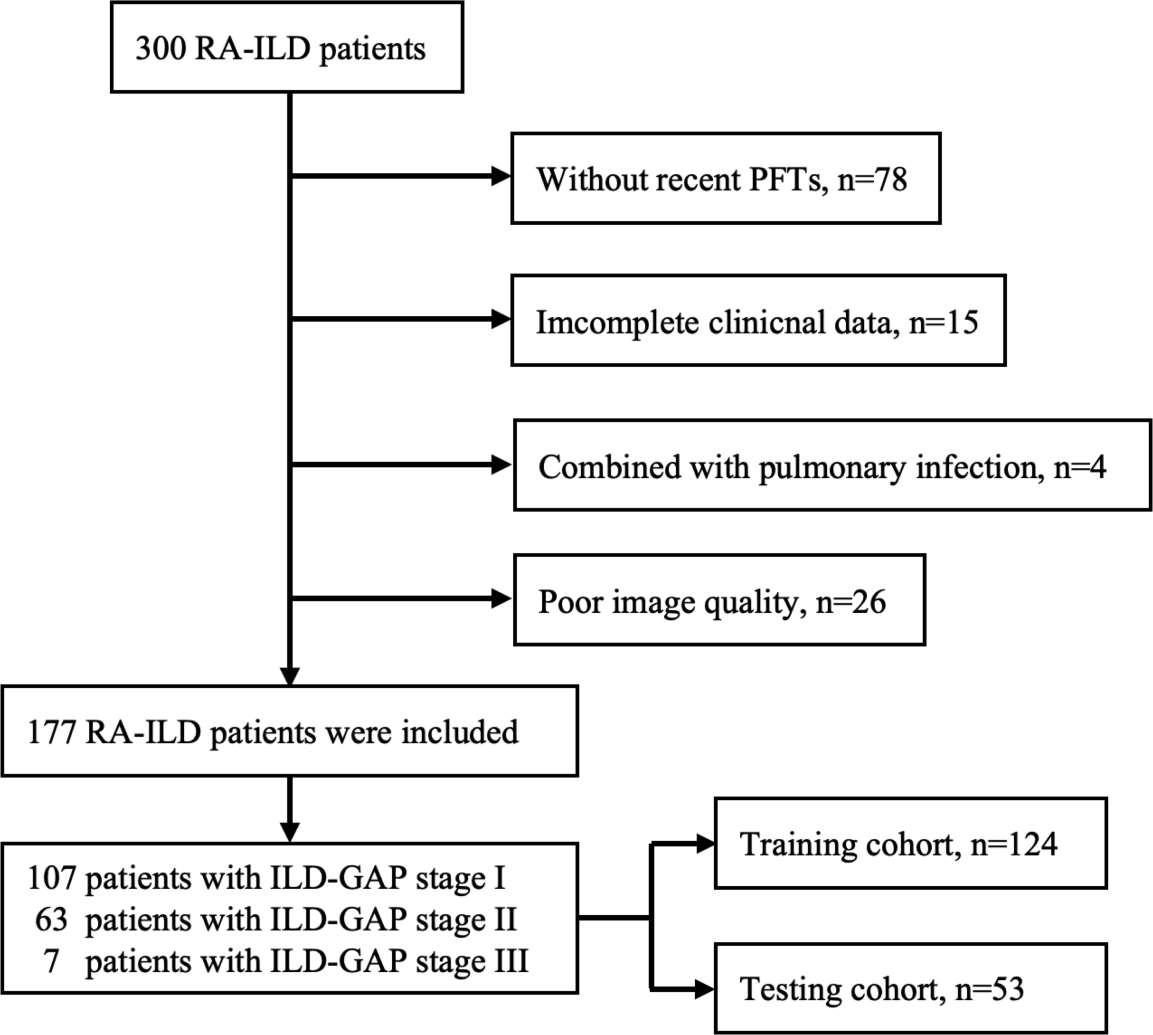
Flowchart of the patient cohort.

The study included patients clinically diagnosed with RA-ILD between April 2020 and December 2023 at Guanghua Hospital Affiliated with Shanghai University of Traditional Chinese Medicine. Inclusion criteria comprised patients meeting all of the following conditions: 1) diagnosed with RA according to the 2010 American College of Rheumatology criteria for RA (24); 2) diagnosed with ILD according to the American Thoracic Society, European Respiratory Society, Japanese Respiratory Society, and Latin American Thoracic Society (ATS/ERS/JRS/ALAT) criteria for ILD (25); 3) underwent a CT scan showing signs of ILD within 3 months after clinical diagnosis; and 4) underwent pulmonary function tests and laboratory examination within 30 days before or after the CT scan. Exclusion criteria were applied for patients meeting any of the following conditions: 1) those with pulmonary edema, infection, drug toxicity, allergy tumor, or heart disease; 2) diagnosed with a combination of other types of CTD; 3) incomplete demographic or clinical data. The flowchart of the study subjects is shown in **Figure 1**.

### 2.2 Pulmonary function test

The routine PFTs were conducted using the Master Screen Diffusion Pulmonary Function Instrument (Eric Jaeger, Germany). The following indicators were assessed: the percentage predicted values (% predicted) of forced expiratory volume in 1 s (FEV1), FVC, total lung capacity (TLC), and DL_CO_. The ILD-GAP index was calculated in accordance with the method proposed by Ryerson et al. (9). Subsequently, patients were categorized into two groups: Group I comprised patients with an ILD-GAP index ≤1, while Group II included patients with an ILD-GAP index >1.

### 2.3 CT image acquisition and evaluation

All enrolled patients underwent nonenhanced chest CT examinations using one of two multidetector CT systems: SOMATOM Definition Flash (Siemens Healthcare, Tokyo, Japan) or Access CT (Philips Healthcare, Andover, Massachusetts, USA). The parameters used for CT scanning were as follows: tube voltage of 120 kVp and tube current-time product of 60-220 mAs with automatic dose modulation; detector collimation of 64 × 0.6 mm; rotation time of 1.0 second; and matrix size of 512 × 512. All CT scans were reconstructed with a 1-mm slice thickness and lung convolution kernels. The semiquantitative CT (SQCT) assessment was carried out to calculate Goh score for each CT scan (26). RA-ILD findings from HRCT were classified as UIP or non-UIP patterns following recent IPF guidelines (25).

### 2.4 Three-dimensional lung segmentation

All image segmentation was executed using 3D Slicer software (version 5.6.1, www.slicer.org). The preprocessing steps were carried out as follows: 1) All CT images were reprocessed using the “Resample Scalar Volume” module by resampling them into 1-mm thick slices and normalizing the intensity values within the range of [–1, 1]. 2) Using the “Radiomics” module, the voxel intensity values were discretized with a fixed bin width of 25 HU to reduce noise and standardize intensity across the images. 3) Z-score normalization was performed on the image gray values to reduce the impact of inconsistent imaging parameters on the variability of radiomics features. 4) The region of interest (ROI) of the bilateral lungs was automatically segmented, encompassing blood vessels and the trachea in the lung lobes (window width = 1,250; window level = -875). A threshold-based region growing method was utilized. The seeding strategy involved the placement of a total of 13 seed points across different anatomical planes. On the axial plane, three seed points were positioned in the peripheral regions of the left and right lungs, respectively. A similar approach was adopted on the coronal plane. Additionally, one seed point was positioned at the location of the main bronchus. Subsequently, the segmentation results underwent manual correction by a radiologist with 5 years of experience in imaging diagnosis of chest diseases, and confirmation was obtained from another radiologist with 8 years of experience in imaging diagnosis of chest diseases.

Interclass and intraclass correlation coefficients (ICCs) were employed in the following manner: A total of 20 cases were randomly selected for region of interest (ROI) segmentation by Radiologist 1. Radiologist 2 then replicated the segmentation for these 20 cases. Subsequently, Radiologist 1 repeated the segmentation after a one-month interval. The segmentation was deemed well-matched in terms of interobserver reliability and intraobserver reproducibility when the ICC value surpassed 0.75.

### 2.5 Radiomics feature extraction and model establishment

**Figure 2:**
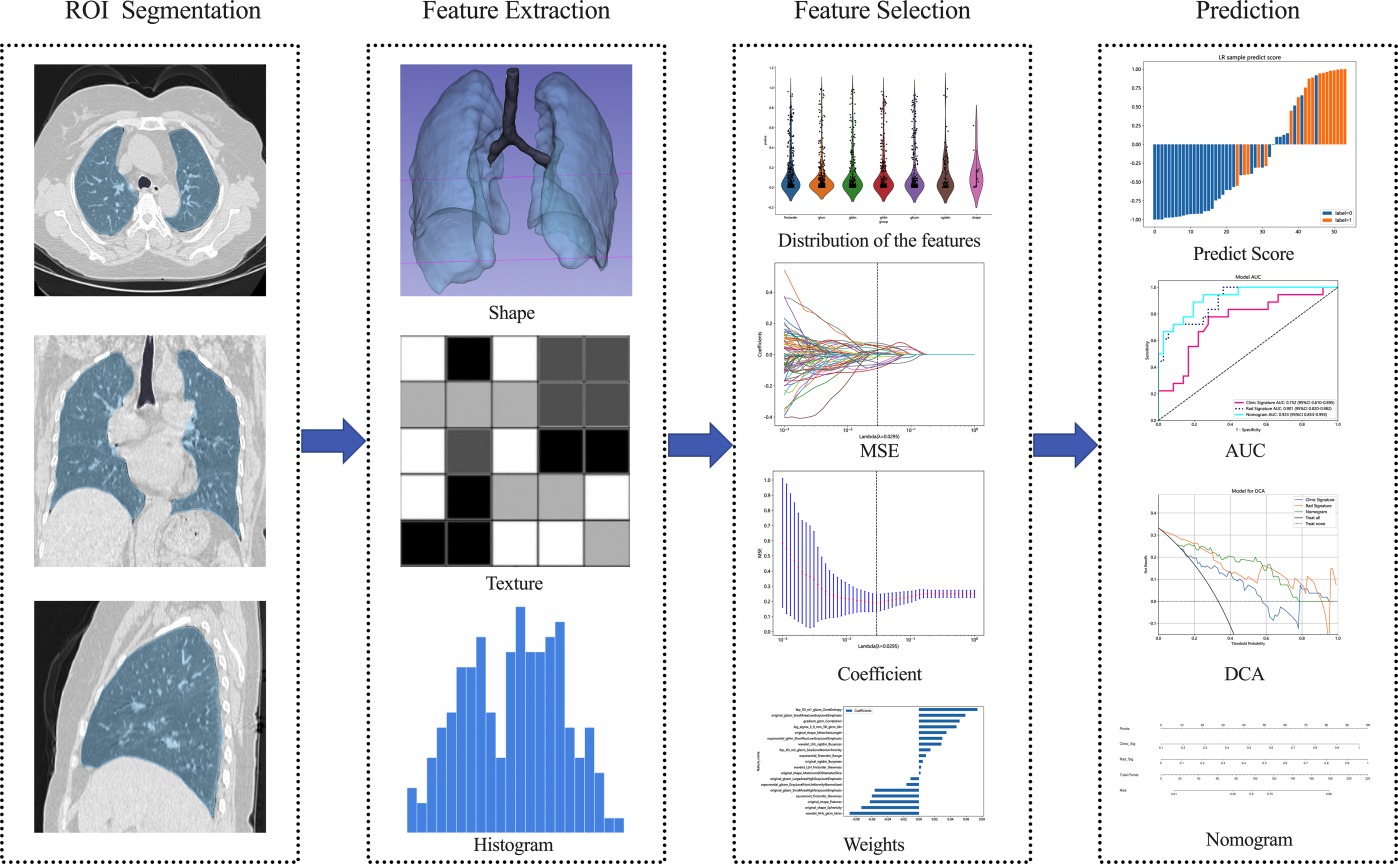
Overview workflow of radiomics analysis. Semi-automatic segmentation of the whole lung was performed on CT images, followed by manual adjustment of the confirmed dissection range, with the region of interest delineated in blue. Imaging-derived histologic features, including shape and texture characteristics, were extracted from CT images of both lungs. Feature selection was conducted using inter- and intra-observer reliability assessment as well as the LASSO method. The performance and clinical utility of predictive model were evaluated using ROC, DCA, and nomogram analysis. MSE Mean standard error, ROC Receiver operating characteristic, DCA Decision curve analysis.

**Figure 2** shows the workflow of radiomics analysis in this study. The patient cohort was randomly split into training and test cohorts at a ratio of 7:3. Feature extraction was performed utilizing the open-source Pyradiomics software package (http://pypi.org/project/pyradiomics/). This package facilitates the extraction of a comprehensive suite of radiomics features, categorized into seven distinct classes: Gray Level Dependence Matrix (GLDM), Gray Level Co-occurrence Matrix (GLCM), Gray Level Run Length Matrix (GLRLM), Gray Level Size Zone Matrix (GLSZM), Neighboring Gray Tone Difference Matrix (NGTDM), First Order Statistics, and Shape-based features (3D). A detailed description of the extracted features is accessible via the Pyradiomics documentation (http://pyradiomics.readthedocs.io). A total of 1,834 radiomics features were extracted from the ROIs. Statistical analysis involved the Student’s t-test for normally distributed features and the Mann-Whitney U test for others. Features with a p-value ≤ 0.05 were retained, resulting in 1,171 features. Spearman’s rank correlation coefficient was then applied to identify robustly repeatable features, retaining one feature from pairs with a correlation coefficient > 0.75. A recursive elimination strategy further refined the features to a subset of 102. The dataset’s signature was constructed using the least absolute shrinkage and selection operator (LASSO) regression model. The optimal λ value was determined via tenfold cross-validation. Features with non-zero coefficients formed the Radiomics Signature, combining linearly to compute the radiomics score for each patient. Scikit-learn in Python was employed for LASSO regression, and logistic regression was used for model formulation after 10-fold cross-validation to verify model adequacy.

### 2.6 Construction of the clinical model

The clinical factor model incorporated variables that were significantly different (*p* < 0.05) as determined by univariate logistic regression analysis. These variables included clinical data and laboratory examinations from the training cohort. Odds ratios (ORs) with 95% confidence intervals (CIs) were calculated for the significantly correlated variables. To mitigate the risk of data leakage within the models, gender, age, and PFT parameters were excluded.

### 2.7 The building of the clinical-radiomics nomogram

A multivariate logistic regression analysis, combining both the clinical signature and radiomics signature, was employed in a backward step-down selection procedure to develop the final integrated radiomics-clinical prediction model.

### 2.8 Statistical analysis

Statistical analyses were performed using SPSS (version 26.0; IBM Corp.). Statistical significance was defined as a two- sided *p*-value ≤ 0.05. Normally distributed data were analyzed using independent T-tests, and non-normally distributed data were presented as medians (interquartile range) using Mann-Whitney U tests. Categorical variables were analyzed using chi-square tests. The predictive performance of the three models was evaluated using receiver operating characteristic (ROC) curves, with the area under the ROC curve (AUC) calculated. Model performance was tested in both the training and test cohorts. The Delong test was applied to compare AUCs among the three models. Calibration efficiency of the nomogram was assessed through calibration curves, and the Hosmer–Lemeshow analytical fit was used to evaluate calibration ability. Decision curve analysis (DCA) was employed to evaluate the clinical utility of the radiomics-clinical model.

## 3 Results

### 3.1 Patient characteristics

A total of 177 patients with RA-ILD were enrolled in this study. Among these patients, 107, 63, and 7 were allocated to ILD-GAP stage I, II, and III, respectively. To prevent excessive data bias, the patients in ILD-GAP stage II and III were combined into a single group. **Table 1** listed the baseline patient characteristics in group I and group II. Age, gender, FVC, FEV1, TLC, DL_CO_, and serum Krebs von den Lungen-6 (KL-6) level showed significant differences (*p* < 0.05) between the two groups, while the differences in smoking history, ACPA, RF-IgM, RF-IgA, and RF-IgG were not significant (*p* > 0.05). In addition, there was no significant statistical difference between the two groups in terms of ESR, CRP, TNFα, IFNγ, IFNα, as well as disease activity score (*p* > 0.05).

**Table 1:** Patient characteristics.

Variables	Group I (n=107)	Group II (n=70)	*p* value
Female (%)	91(85.05%)	40(57.14%)	<0.001
Age, years	58.8 ± 8.9	71.5 ± 5.5	<0.001
RA duration, years	10.00 [4.00-9.25]	11.00 [4.00-20.00]	0.084
Smoking (%)	7(6.54%)	5(7.14%)	0.876
Lung function			
FVC%	86.5 ± 18.1	66.3 ± 17.4	<0.001
FEV1%	86.2 ± 18.0	67.2 ± 17.0	<0.001
TLC%	83.6 ± 15.7	56.8 ± 14.6	<0.001
DL_CO_%	61.5 ± 17.7	32.2 ± 13.4	<0.001
Laboratory Examinations			
ACPA, RU/ml	653.90 [240.30-1249.50]	582.10 [138.75-1364.88]	0.782
RF-IgA, U/ml	32.77 [8.22-300.00]	28.18 [6.43-146.40]	0.496
RF-IgG, U/ml	30.01 [6.11-96.76]	40.50 [4.23-136.63]	0.957
RF-IgM, U/ml	127.00 [33.90-369.00]	135.00 [40.25-574.00]	0.590
TNFα, pg/ml	2.56 [1.68-2.67]	2.00 [1.36-2.56]	0.075
IFNγ, pg/ml	2.46 [2.27-5.65]	2.46 [1.82-5.05]	0.745
IFNα, pg/ml	1.36 [0.95-2.09]	1.50 [0.96-1.88]	0.830
ESR, mm/h	37.50 [23.75-65.25]	40.00 [18.00-69.00]	0.682
CRP, mg/l	12.35 [2.06-32.95]	7.14 [0.80-22.98]	0.197
KL-6, U/ml	216.58 [137.09-297.30]	376.84 [261.07-539.88]	<0.001
Disease activity			
DAS-28-ESR	3.51 ± 1.56	3.33 ± 1.37	0.489
DAS-28-CRP	4.25 ± 1.54	4.12 ± 1.46	0.611
CT images			
ILD pattern (UIP/non-UIP)	50 (46.7%)	64.3(64.3%)	0.022
Goh score, %	12 [8-15]	19 [13-27]	<0.001
Treatment for RA			
Methotrexate	75 (72.8%)	45 (66.2%)	0.353
Methylprednisolone	47 (46.5%)	37 (57.8%)	0.158
Hydroxychloroquine	18 (18.2%)	11 (16.4%)	0.769
Leflunomide	20 (19.8%)	18 (26.9%)	0.284
Biological agent	69 (67.0%)	30 (44.8%)	0.004

Categorical variables are presented as n (%). Continuous variables are listed as median (inter-quartile range, IQR) or as mean ± standard deviation. n, number of patients; FVC, Forced vital capacity; FEV1, Forced expiratory volume in 1 s; TLC, Total lung capacity; DLCO, Diffusion capacity for carbon monoxide; ESR, erythrocyte sedimentation rate; RF, rheumatoid factor; CRP, C-reactive protein; APLA, anti-phospholipid antibodies; KL-6, Krebs von den Lungen-6; TNFα, tumor necrosis factor alpha; IFNγ, interferon gamma; IFNα, interferon alpha; DAS, disease activity score; UIP, usual interstitial pneumonia.

### 3.2 Development of the clinical model

Univariate logistic regression was performed to analyze the clinical data and laboratory examinations (**Table 2**). To ensure the reliability of the model construction, factors such as gender, age, and PFT parameters were excluded. Then, KL-6 (ORs = 1.007; 95% CI, 1.004-1.010; *p* < 0.001) was selected as independent clinical risk factors.

**Table 2:** Independent risk factors in training cohort.

Variables	Odds ratio (95% CI)	*p* value
Age	1.27(1.17-1.38)	<0.001
Gender	0.30(0.13-0.69)	0.005
RA duration	1.03(1.00-1.07)	0.068
FVC%	0.91(0.88-0.94)	<0.001
FEV1%	0.94(0.92-0.97)	<0.001
TLC%	0.89(0.85-0.92)	<0.001
DL_CO_%	0.88(0.84-0.92)	<0.001
ACPA	1.000(0.996-1.004)	0.936
RFIgM	1.000(0.998-1.001)	0.678
RFIgG	1.000(0.996-1.004)	0.936
RFIgA	0.998(0.995-1.002)	0.347
KL-6	1.007(1.004-1.010)	<0.001
TNFα	1.02(0.97-1.07)	0.457
IFNα	1.04(0.94-1.15)	0.419
IFNγ	0.99(0.91-1.07)	0.771
CRP	0.99(0.97-1.00)	0.183
ESR	0.99(0.97-1.00)	0.168
DAS-28-CRP	0.84(0.64-1.11)	0.219
DAS-28-ESR	0.85(0.65-1.11)	0.227

CI, confidence-interval; ORs, Odds ratio; FVC, Forced vital capacity; FEV1, Forced expiratory volume in 1 s; TLC, Total lung capacity; DLCO, Diffusion capacity for carbon monoxide; ESR, erythrocyte sedimentation rate; RF, rheumatoid factor; CRP, C-reactive protein; APLA, anti-phospholipid antibodies; KL-6, Krebs von den Lungen-6, TNFα, tumor necrosis factor alpha; IFNγ, interferon gamma; IFNα, interferon alpha; DAS, disease activity score.

### 3.3 Development of the radiomics model

**Figure 3:**
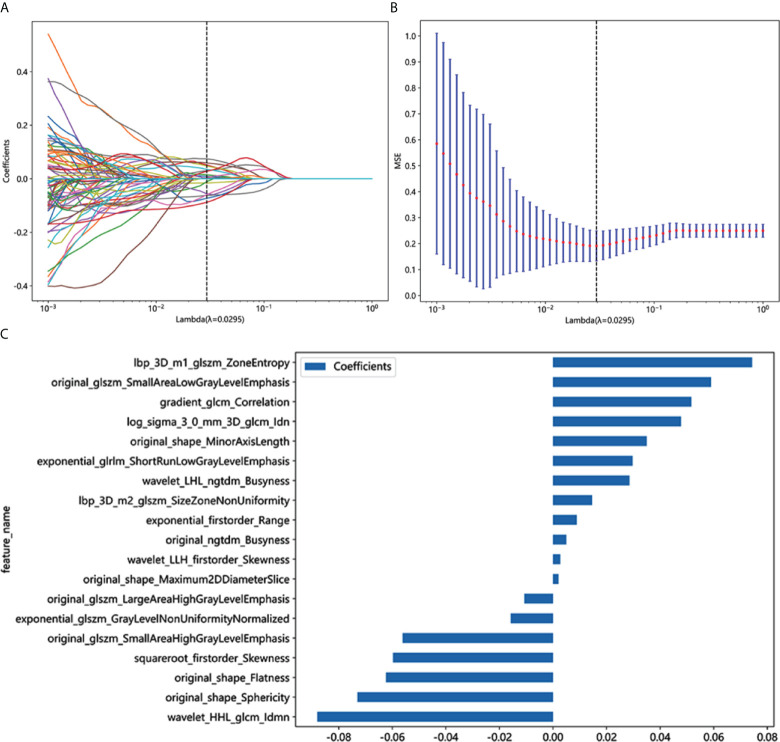
Radiomics feature selection based on LASSO algorithm and Rad score establishment. **(A)** LASSO coefficient profile plot with different log (λ)was shown. **(B)** Ten-fold cross-validated coefficients and 10-fold cross-validated MSE. **(C)** The histogram of the Rad score based on the selected features.

A total of 1,834 radiomics features were extracted from the CT images, with 1,171 exhibiting promising interobserver and intraobserver agreement (intraclass correlation coefficient > 0.75). Through LASSO logistic regression analysis, 102 significantly different (*p* < 0.05) radiomics features were selected to identify optimally related features. Ultimately, 19 features were included in the construction of the radiomics model. **Figures 3A,B** show the coefficients and mean standard error (MSE) for the 10-fold validation, while **Figure 3C** presents the coefficient values for the final selection of non-zero features Rad score is shown as follows: Rad-score= 0.4227 + 0.0088 × exponential_firstorder_Range +0.0296 × exponential_glrlm_ShortRunLowGrayLevelEmphasis -0.0157 × exponential_glszm_GrayLevelNonUniformityNormalized +0.0516 × gradient_glcm_Correlation +0.0743 × lbp_3D_m1_glszm_ZoneEntropy +0.0146 × lbp_3D_m2_glszm_SizeZoneNonUniformity +0.0477 × log_sigma_3_0_mm_3D_glcm_Idn -0.0107 × original_glszm_LargeAreaHighGrayLevelEmphasis -0.0561 × original_glszm_SmallAreaHighGrayLevelEmphasis +0.0590 × original_glszm_SmallAreaLowGrayLevelEmphasis +0.0049 × original_ngtdm_Busyness -0.0623 × original_shape_Flatness +0.0020 × original_shape_Maximum2DDiameterSlice +0.0349 × original_shape_MinorAxisLength -0.0730 × original_shape_Sphericity -0.0597 × squareroot_firstorder_Skewness -0.0879 × wavelet_HHL_glcm_Idmn +0.0285 × wavelet_LHL_ngtdm_Busyness +0.0026 × wavelet_LLH_firstorder_Skewness.

### 3.4 Comparison of clinical, radiomics, and nomogram models

**Figure 4:**
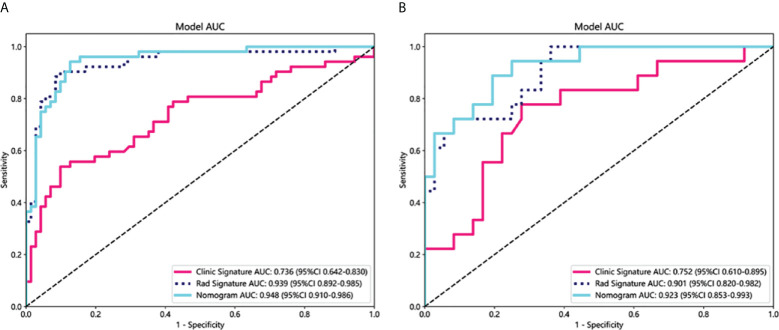
Comparison of receiver operating characteristic (ROC) curves for the clinical, radiomics, and nomogram models in the training **(A)** and testing **(B)** cohorts. The combined nomogram performed optimally in both the training and testing cohorts.

**Figure 5:**
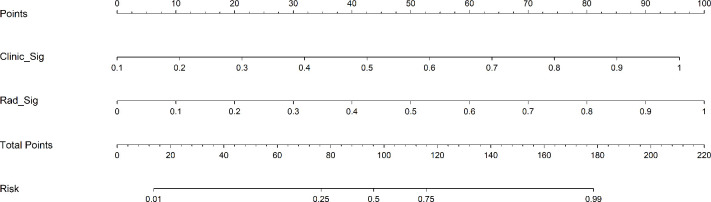
A constructed nomogram for predicting the GAP staging of RA-ILD.

As shown in **Figure 4**, for the AUC, the clinical features [0.736, 95%CI = 0.642–0.830) and the radiomics features (0.939, 95%CI = 0.892–0.985) were perfectly fitted for the training cohort. In the testing cohort, the clinical characteristics (0.752, 95%CI = 0.610–0.894) and the radiomics signature remained well-fitted (0.901, 95%CI = 0.820–0.982). As shown in **Figure 5**, The nomogram using the LR algorithm, combining clinical features and radiomics features, showed the best performance in the training (0.948, 95%CI = 0.910–0.987) and testing cohort (0.923, 95%CI = 0.853–0.993), respectively. The detailed diagnostic efficiency capability for each model is presented in **Supplementary Table S1**.

To compare the clinical signature, radiomics signature, and nomogram, the Delong test was utilized (**Supplementary Table 2**). In the testing cohort, the results indicated that the AUC comparison between the nomogram and the clinical signature achieved 0.021, suggesting that the nomogram outperformed the clinical model in discriminating the GAP staging of RA-ILD. The AUC comparison between the nomogram and radiomics signature was 0.219, indicating that both models performed well in differentiating the GAP staging of RA-ILD.

### 3.5 Comparison of visual assessment, radiomics, and nomogram models

In the testing cohort, the Goh score achieved an AUC of 0.820 (95%CI=0.700-0.941; **Supplementary Figure 1**). Comparatively, both the radiomics model (0.901, 95% CI: 0.820-0.982) and the combined radiomics-KL-6 nomogram model (0.923, 95% CI: 0.853-0.993) showed superior AUC values relative to the Goh score.

### 3.6 Calibration curve and DCA of the models

**Figure 6:**
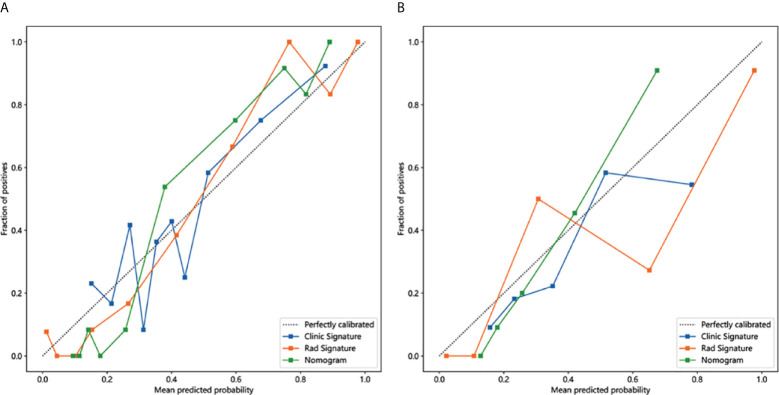
Calibration curves in the training and testing cohorts showing that the nomogram fits perfectly well in both the training **(A)** and testing cohorts **(B)**.

The calibration curves for the training and testing cohorts were shown in **Figure 6**. The p-values from the Hosmer-Lemeshow test for clinical features, radiologic features, and nomograms were 0.557, 0.171, 0.305, and 0.193, 0.072, 0.160 in the training and test cohorts, respectively. These p-values suggest a perfect agreement for each model (**Supplementary Table 3**).

**Figure 7:**
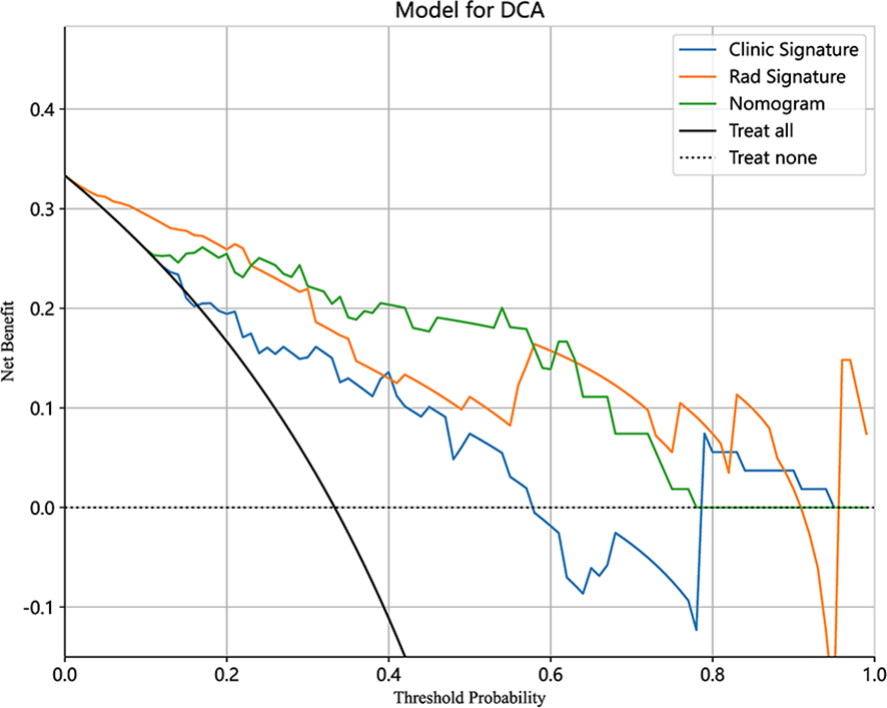
Decision curve analysis (DCA) of the clinical, radiomics, and nomogram models in the testing cohort.

As shown in **Figure 7**, the DCA for clinical features, radiographic features, and nomograms, covering predictive probabilities from 0.12 to 0.41, 0.02 to 0.91, and 0.1 to 0.78. The nomogram achieves the largest net benefit compared to other models when the threshold probability ranges from 0.23 to 0.58.

## 4 Discussion

In our study, the radiomics model based on chest CT has great performance to distinguish different ILD-GAP stage patients with an AUC of 0.901 in validation cohort. The nomogram model, combining the radiomics model and serum KL-6, further enhanced the prediction efficiency of GAP staging with an AUC of 0.948 and 0.923 in the training and validation cohort, respectively.

Among the serological markers, anti-citrullinated protein antibodies (ACPA) have been implicated in the extra-articular manifestations of RA, including ILD (27–29). Correia et al. reported a correlation between ACPA titers and the risk of developing ILD (30). On the contrary, many studies have shown no association between ACPA and ILD, as well as related RF factors. Similarly, our study revealed no significant differences between ACPA and RF factors in different stages of RA-ILD. However, these different results may be attributed to the heterogeneity of ACPA specificity and search methods (5, 31). It is worth noting that treatment strategies may play a crucial role in the development and progression of RA-ILD. A higher proportion of biological agent use was revealed in the low-risk group by our analysis. This suggests that patients using biological agents may represent a cohort receiving early and aggressive treatment. The use of biological agents may interrupt the inflammatory cascade leading to ILD, thereby reducing the risk of developing severe ILD in later stages (32, 33).

In addition, older age and male sex have been strongly associated with RA-ILD (34). We excluded gender, age, and PFTs parameters from the clinical model to prevent data leakage, despite their status as independent risk factors. Eventually, univariate logistic regression analysis revealed that KL-6 was an independent predictor in our present study. KL-6 is a mucin-like glycoprotein which stimulates fibrosis and inhibits apoptosis of pulmonary fibroblasts (35, 36). Elevated serum KL-6 levels have been observed in RA patients with lung involvement, suggesting its potential utility in early detection of ILD. In a cohort of 50 RA patients, KL-6 levels positively correlated with the high-resolution computed tomography fibrosis score, indicating that high KL-6 levels are a significant biomarker for ILD and may serve as a predictor for ILD severity in RA patients (37). Moreover, a study suggests that high KL-6 levels might be an independent risk factor and useful for the prognosis in patients with RA-ILD (38). So far, the utility of serum KL-6 has been evaluated in several forms of ILD and its sensitivity and specificity for RA-ILD ranged from 67%-85% and 60%-90%, respectively, depending on the cutoff value (36, 37, 39). In our study, a clinical factor model to classify RA-ILD stages was developed based on KL-6, and then achieved an AUC of 0.752 in the testing cohorts.

Radiomics is an objective technique offering a reliable and comprehensive quantitative assessment of images, unaffected by inter-reader variability (40). Feature extraction involves mathematical operations on digital images to generate numerical descriptors of texture, shape, and other distinct characteristics. These descriptors can be computationally analyzed to explore potential associations with clinical parameters (41). Particularly useful for diseases challenging to describe through simple visual features, high-dimensional abstract features extracted from wavelet-transformed images can provide diverse perspectives in capturing hidden information not easily observed visually. Radiomics features have indeed proven their potential for severity estimation in Systemic sclerosis-ILD and guiding treatment decisions (42). At present, the literature on the application of radiomics is limited. Venerito et al. (23) retrieved the HRCTs of 30 RA-ILD patients and suggested that radiomics analysis could predict patient mortality. This finding suggests that HRCT could serve as a digital biomarker for RA-ILD, offering prognostic value that is independent of the clinical characteristics of the disease. Recently, some scholars have developed radiomics models based on CT images to differentiate GAP staging in CTD patients. Qin et al. (21) manually segmented the right lung of CTD-ILD patients and constructed a radiomics model from the 9 extracted texture features. The AUC of their radiomics models in the validation cohort was 0.787 and 0.718 in the internal and external test cohort, respectively. A similar study utilized a semi-automatic segmentation method to segment bilateral lungs, obtaining a total of 4 features (22). Their developed radiomics model demonstrated an AUC of 0.801 in the test cohort. Instead of focusing on all types of CTDs, we concentrated on patients with RA. In our work, totally1,834 radiomics features obtained from the CT images, 19 higher-order texture features extracted from wavelet transformed images were acquired as remarkable elements to build the radiomics model, resulting in an AUC of 0.939, and 0.901 in the training and testing cohorts, respectively. It is speculated that by targeted with ILD specifically caused by RA, to some extent excluded the imbalance of training data arising from the heterogeneous imaging characteristics of various CTD-ILD subtypes (43), which eventually screened out more features. In the current study, we constructed a nomogram model that integrates the radscore with serum KL-6 levels to further enhance the accuracy of predicting low-risk RA-ILD. In contrast to the GAP index, the nomogram model can predict GAP staging in patients with RA-ILD even when precise lung function parameters are challenging to obtain. This radiomics-based approach may serve as a supportive tool for assessing the severity of RA-ILD. Moreover, the proposed model can be readily implemented in clinical practice, as it leverages routinely acquired chest CT imaging and serum biomarker data to automate the computational process, thereby minimizing the operational burden on clinicians.

There are certain limitations in our study. Firstly, the single-center design with a relatively small overall sample size, especially the limited representation of more severe ILD-GAP stage III patients, may restrict the model ability. Future studies based on larger datasets from other centers are needed to evaluate model generalizability. Secondly, the exact mortality of the retrospective study verified by the GAP index system may less precise than actual mortality of patient. Nevertheless, as an available method to predict mortality, the GAP index system has been validated in RA-ILD. The precise assessment of mortality risk will be conducted in our further research. In addition, our study serves as a foundational exploration, offering valuable insights for selecting valuable imaging biomarkers in RA-ILD.

In conclusion, a novel nomogram model combining CT-based radiomics and serum KL-6 was developed in our study. It shows good prediction accuracy in predicting low-risk RA-ILD patients, which implies that this noninvasive and quantitative method may impact the clinical decision-making process, offering a more precise management strategy for patients with RA-ILD.

## Data availability statement

The original contributions presented in the study are included in the article/**Supplementary Material**. Further inquiries can be directed to the corresponding authors.

## Ethics statement

The study protocol was approved by the Ethics Committee of Guanghua Hospital Affiliated to Shanghai University of Traditional Chinese Medicine (2023-K-46). The studies were conducted in accordance with the local legislation and institutional requirements. Written informed consent for participation in this study was provided by the participants’ legal guardians/next of kin.

## Author contributions

NH: Writing – original draft. ZG: Writing – original draft. DZ: Writing – original draft. YZ: Writing – original draft. YQ: Writing – original draft. GL: Writing – original draft. XG: Conceptualization, Supervision, Writing – review & editing. LJ: Conceptualization, Supervision, Writing – review & editing.
