# Supplementary material for: A nomogram model combining computed tomography-based radiomics and Krebs von den Lungen-6 for identifying low-risk rheumatoid arthritis-associated interstitial lung disease
Source: Front Immunol. 2024 Aug 1;15:1417156. doi: 10.3389/fimmu.2024.1417156 (PMC11324433; doi:10.3389/fimmu.2024.1417156)
Supplement: Supplementary file 1 [file DataSheet_1.docx]

Supplementary Material

# Supplementary Table1

| **Supplementary Table S1. Capability of the diagnostic efficiency for each model** | | | | | | | |
| --- | --- | --- | --- | --- | --- | --- | --- |
| Model | AUC (95% CI) | Sensitivity | Specificity | Accuracy | PPV | NPV | F1score |
| Clinical Model (Training) | 0.736(0.642-0.830) | 0.519 | 0.901 | 0.74 | 0.794 | 0.719 | 0.628 |
| Clinical Model (Testing) | 0.752(0.610-0.895) | 0.722 | 0.722 | 0.722 | 0.565 | 0.839 | 0.634 |
| Radiomics Model (Training) | 0.939(0.892-0.985) | 0.885 | 0.901 | 0.894 | 0.868 | 0.914 | 0.876 |
| Radiomics Model (Testing) | 0.901(0.820-0.982) | 0.667 | 0.917 | 0.833 | 0.8 | 0.846 | 0.727 |
| Nomogram Model (Training) | 0.948(0.919-0.987) | 0.923 | 0.873 | 0.894 | 0.842 | 0.939 | 0.881 |
| Nomogram Model (Testing) | 0.923(0.853-0.993) | 0.833 | 0.806 | 0.815 | 0.682 | 0.906 | 0.75 |
| PPV, positive predictive value; NPV, negative predictive value. | | | | | | | |

# Supplementary Table2

| **Supplementary Table S2. Delong test of each model** | | | |
| --- | --- | --- | --- |
| Cohort | Clinical model Vs Radiomics model | Nomogram Vs Clinical model | Nomogram Vs Radiomics model |
| Train | <0.001 | <0.001 | 0.269 |
| Test | 0.072 | 0.021 | 0.219 |

# Supplementary Table3

| **Supplementary Table S3. Hosmer–Lemeshow test of each model** | | | |
| --- | --- | --- | --- |
| Cohort | Clinic model | Radiomics model | Nomogram |
| Train | 0.557 | 0.171 | 0.305 |
| Test | 0.193 | 0.072 | 0.16 |

# Supplementary Figure1


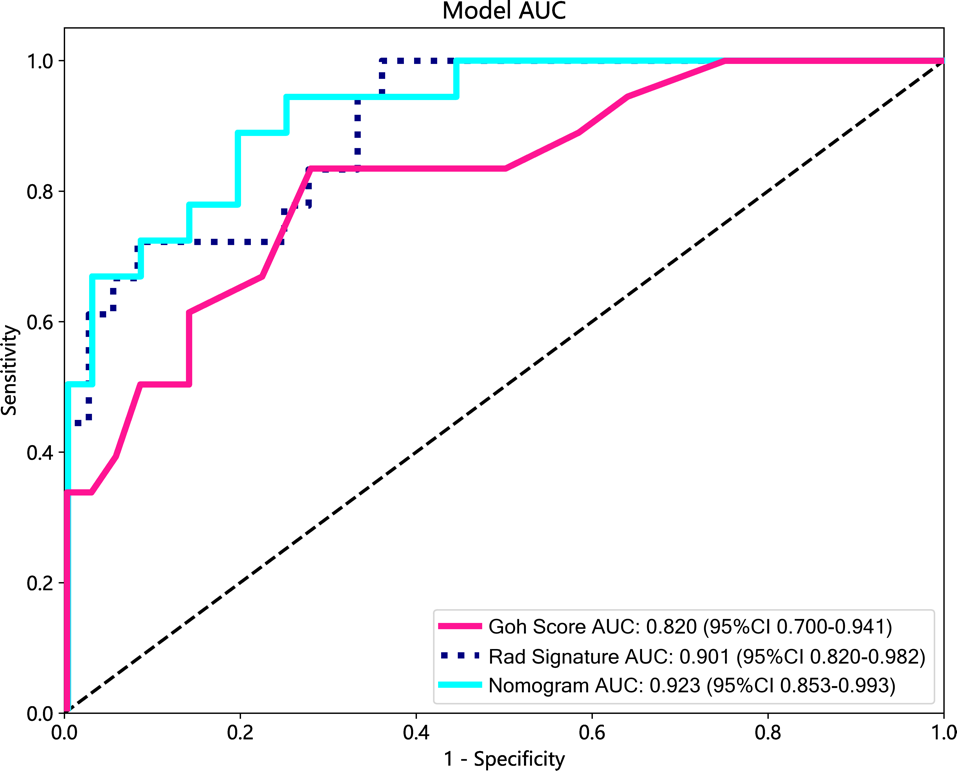


**SUPPLEMENTARY FIGURE 1** Comparison of receiver operating characteristic (ROC) curves for visual assessment, radiomics, and nomogram models in the testing cohorts.
